# 982. A Comparison of Decolonization Methods and Impact on MRSA Infection in a Neonatal Intensive Care Unit

**DOI:** 10.1093/ofid/ofad500.037

**Published:** 2023-11-27

**Authors:** Nahid Hiermandi, Catherine Foster, Krystal Purnell, Judith R Campbell, Lucila Marquez

**Affiliations:** Baylor College of Medicine, Houson, TX; Baylor College of Medicine, Houson, TX; Texas Children's Hospital, Houston, Texas; Baylor College of Medicine, Houson, TX; Baylor College of Medicine, Houson, TX

## Abstract

**Background:**

Infection due to methicillin-resistant *Staphylococcus aureus* (MRSA) causes morbidity and mortality in premature hospitalized neonates. MRSA colonization is a risk factor for transmission and outbreaks within neonatal intensive care units. Decolonization aims to stop MRSA transmission and infection. National guidelines lack recommendations for which decolonization practices are the most effective in neonates. We hypothesize that both topical mupirocin and chlorhexidine (CHG) is more effective than mupirocin alone at preventing MRSA infection in neonates.

**Methods:**

In this retrospective study, we compared the rates of MRSA infection among all colonized neonates admitted to a 42 bed NICU at Texas Children’s Hospital from 2012 to 2020. Our surveillance and decolonization protocol (Figure 1) was introduced in October 2016. The MRSA screening methodology initially consisted of culture, then was changed to PCR (reflexed to culture if MRSA was detected). We grouped colonized neonates as those who underwent no decolonization, partial decolonization (mupirocin only), and full decolonization (CHG+ mupirocin) and compared the rates of infection in each. Infection was defined by isolation of MRSA from any specimen in the context of illness. This study was approved through the Baylor College of Medicine Institutional Review Board.

Figure 1

**Figure:**
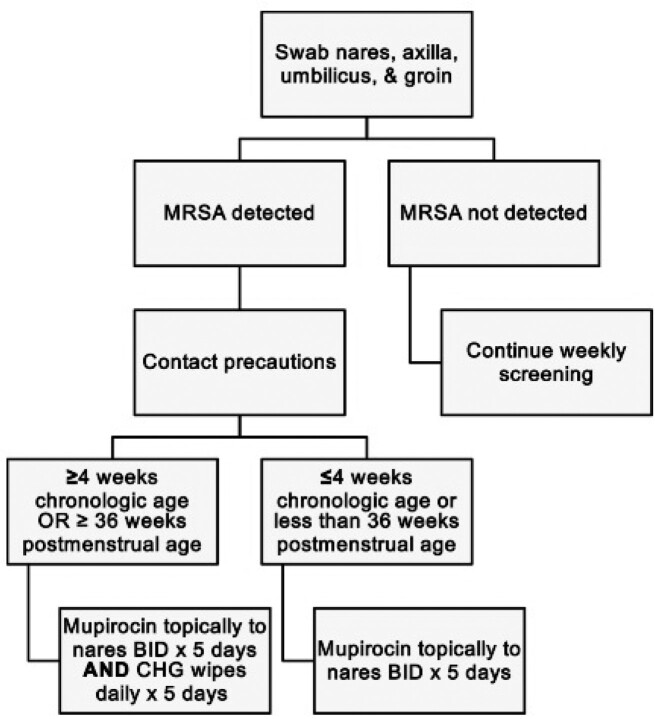

MRSA surveillance and decolonization protocol

**Results:**

7,980 neonates were admitted to the NICU from 2012-2020. Of those screened, 128 were MRSA-colonized. Eighteen were fully decolonized, 76 partially decolonized and 34 received neither (Table 1). Zero infants who underwent full decolonization had MRSA infection. Decolonized infants were less likely to develop infection (OR 0.56, 95% C.I. 0.2-1.58) compared to infants who did not undergo any decolonization. Neonates who received only mupirocin (due to age) were less likely to develop infection (OR 0.72, 95% C.I. 0.26-2.04), however this reduction was not as impactful as with the combination of CHG and mupirocin (Figure 2).

Table 1

**Figure:**
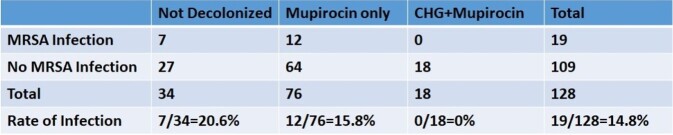

MRSA infection rates by category of decolonization

Figure 2

**Figure:**
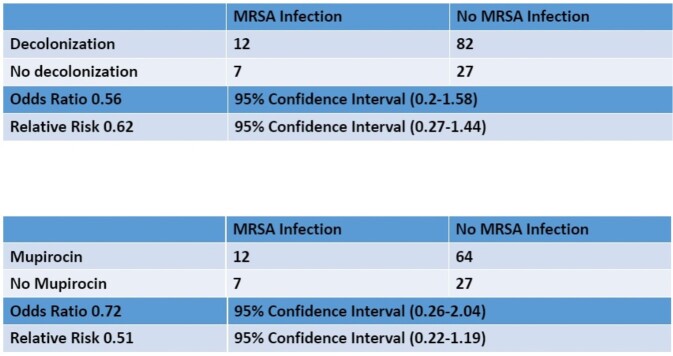

Comparison of MRSA infection between decolonized vs not decolonized and mupirocin vs no mupirocin with odds ratio and relative risk

**Conclusion:**

Neonates who received CHG and mupirocin did not develop MRSA infection, and decolonization with mupirocin alone provides some but not as much protection from MRSA disease as does both mupirocin and CHG. Further studies should evaluate the safety of CHG wipes in neonates less than 36 weeks gestation/4 weeks chronologic age.

**Disclosures:**

**All Authors**: No reported disclosures

